# Slow-Release Sachets of *Neoseiulus cucumeris* Predatory Mites Reduce Intraguild Predation by *Dalotia coriaria* in Greenhouse Biological Control Systems

**DOI:** 10.3390/insects6020489

**Published:** 2015-06-01

**Authors:** Emily Pochubay, Joseph Tourtois, Jeanne Himmelein, Matthew Grieshop

**Affiliations:** 1 Michigan State University, 6686 S. Center Hwy, Traverse City, MI 49684, USA; E-Mail: pochubay@anr.msu.edu; 2 Michigan State University, 205 Center for Integrated Plant Systems, East Lansing, MI 48824, USA; E-Mail: riddlej2@msu.edu; 3 Michigan State University, 3299 Gull Road Floor 4, Nazareth, MI 49074, USA; E-Mail: jeanne@nashgreenhouse.com

**Keywords:** biological control, thrips, greenhouse, intraguild predation

## Abstract

Intraguild predation of *Neoseiulus cucumeris* Oudemans (Phytoseiidae) by soil-dwelling predators, *Dalotia coriaria* Kraatz (Staphylinidae) may limit the utility of open rearing systems in greenhouse thrips management programs. We determined the rate of *D. coriaria* invasion of *N. cucumeris* breeder material presented in piles or sachets, bran piles (without mites), and sawdust piles. We also observed mite dispersal from breeder piles and sachets when *D. coriaria* were not present. *Dalotia coriaria* invaded breeder and bran piles at higher rates than sawdust piles and sachets. Furthermore, proportions of *N. cucumeris* in sachets were six- to eight-fold higher compared with breeder piles. When *D. coriaria* were absent, *N. cucumeris* dispersed from breeder piles and sachets for up to seven weeks. In earlier weeks, more *N. cucumeris* dispersed from breeder piles compared with sachets, and in later weeks more *N. cucumeris* dispersed from sachets compared with breeder piles. Sachets protected *N. cucumeris* from intraguild predation by *D. coriaria* resulting in higher populations of mites. Therefore, sachets should be used in greenhouse biocontrol programs that also release *D. coriaria.* Furthermore, breeder piles that provide “quick-releases” or sachets that provide “slow-releases” of mites should be considered when incorporating *N. cucumeris* into greenhouse thrips management programs.

## 1. Introduction

Biological control tactics for greenhouse arthropod pest management are an economical and attractive alternative to chemical tactics because of greatly lowered risks of phytotoxicity, worker and consumer exposure to harmful chemicals, and development of pest resistance [1]. In greenhouse biological control, multiple predator species are often released to target the spectrum of greenhouse pests inhabiting plant foliage and soil. Natural enemy open rearing systems—*i.e.*, providing natural enemies released in greenhouses with supplemental food or hosts—are used to reduce associated costs of augmentative natural enemy releases by maintaining populations of natural enemies in the greenhouse [2,3,4]. Releasing multiple predators can often result in interactions including competition and intraguild predation that may have positive or negative and direct or indirect effects on predators and pests [5,6,7].

Intraguild predation is common among natural enemies released in greenhouse biological control programs [8,9,10]. Studies observing intraguild predation among natural enemies primarily focus on organisms that occupy similar areas in the crop (e.g., the plant canopy or soil). Some methods for natural enemy release and open rearing place natural enemies in unaccustomed habitats resulting in opportunities for unexpected interactions among predators. For example, *Neoseiulus cucumeris* Oudemans (Phytoseiidae)mites are predators of early instar thrips (Thripidae) that normally inhabit plant canopies [8,11]. Soil-dwelling predators of thrips pupae and shore fly and fungus gnat larvae, *Dalotia coriaria* Kraatz (Staphylinidae) and *Stratiolaelaps miles* (Berlese) (Laelapidae) [12,13,14,15], have been shown to detrimentally impact *N. cucumeris* that are placed on the soil [16]. Intraguild predation has been shown to be especially intense when populations of *N. cucumeris* are maintained using “breeder pile” open rearing systems [16].

Breeder pile open rearing systems are comprised of small (1–3 g) piles of a mixture of bran, *Tyrophagus putrescentiae* (Shrank) (Acaridae) mold mites, and the thrips predatory mites *N. cucumeris*. The bran supports *T. putrescentiae*, an alternative prey for *N. cucumeris*, to maintain the existing *N. cucumeris* and possibly rear new generations of these predatory mites*.* Breeder piles are intended to sustain and produce *N. cucumeris* over multiple weeks thereby reducing the number of *N. cucumeris* releases needed for thrips control. Breeder piles are typically placed onto the soil of potted plants and plug trays. Unfortunately, placing breeder piles on the soil exposes *N. cucumeris* to possible intraguild predation by soil-dwelling predators that feed on *N. cucumeris* and reduce breeder pile productivity of these mites. Therefore, alternative application methods for *N. cucumeris* that reduce the potential for intraguild predation should be investigated.

One means of promoting coexistence of intraguild predators is to increase habitat complexity [6]. Slow-release sachets—paper envelopes—that contain the same mite-bran mixture used to generate breeder piles are an alternative method for releasing mites that may protect *N. cucumeris* from intraguild predators. Furthermore, protecting *N. cucumeris* may influence population dynamics of the mites. These dynamics should be investigated to improve procedures for implementing open rearing systems and maintaining *N. cucumeris* in greenhouses.

Population dynamics of predatory mites in open rearing systems have not been thoroughly investigated. The main focus of studies that have observed predatory mite open rearing systems measure efficacy of management and suppression of pest mites and thrips [11,17,18]. With the exception of Shipp and Wang [11] who measured mite dispersal from slow-release sachets, the production and dispersal rates of predatory mites from breeder piles and slow-release sachets have not been observed. Different dispersal rates of mites may influence timing of releases and appropriate conditions such as optimal pest density and plant maturity for introducing predatory mite open rearing systems. Revealing the temporal production and dispersal of mites from these systems would provide insight on how to optimize the timing of future releases and promote economical release procedures.

We conducted two experiments to address our objectives. In the first study, our objectives were to determine whether *N. cucumeris* sachets hung in the plant canopy prevent *D. coriaria* from entering the mite-bran mixture, and the abundance of *D. coriaria* in breeder piles, bran piles (without mites), sawdust piles, or hanging sachets. We also monitored population dynamics of *N. cucumeris* and mold mites in these treatments. In a second study, our objective was to observe the numbers of mites that dispersed from breeder piles and sachets when *D. coriaria* were not present.

## 2. Experimental Section

### 2.1. Experiment One—Materials and Methods

The first trial of our first experiment was conducted in spring 2011 and a second trial was repeated in fall 2011 in a 1.62 ha certified organic greenhouse at Elzinga and Hoeksema Greenhouses (Portage, MI, USA). The greenhouse temperature and relative humidity were regulated by a Hoogendoorn computer control system (Hoogendoorn Growth Management, The Netherlands) set at 24 °C.

This experiment was a randomized complete block design with five blocks, each block was a 1.68 m × 4.88 m greenhouse bench. Each greenhouse bench was lined with 0.08 mm thick clear polyethylene sheets of plastic with small drainage holes and filled with 13 cm of potting soil mix provided by Morgan Composting (Sears, MI, USA). Certified organic barley (*Hordeum vulgare)* seed from Albert Lea Seed (Albert Lea, MN, USA) and Johnny’s Selected Seeds (Waterville, MA, USA) were broadcast seeded onto the tops of the soil in each of the benches at a rate of 226.8 g per 8.2 m^2^ for the first and second trial, respectively. Barley was grown under natural light for one week before introduction of the randomized treatments, and barley beds were irrigated daily until soil was saturated to a depth of approximately 2.5 cm using an overhead irrigation boom for the duration of the experiment. We selected barley as an experimental habitat for this experiment to prevent unintentional rearing of greenhouse pests.

After one week of barley growth breeder piles, bran piles, sawdust piles, and hanging sachet treatments were introduced in an equally spaced grid pattern onto the greenhouse benches. There were 10 replicates per treatment per block (*i.e.*, greenhouse bench), or a total of 200 experimental units (*n* = 50 per treatment). Breeder piles were produced by measuring and individually placing 60 1.5 g portions (of Amblyseius-Breeding-System (*i.e.*, ABS-System 25,000 provided by BioBest Biological Systems of Ontario, CA, USA) into 59 mL plastic soufflé cups. Breeder pile material contained 277 ± 18.73 (SEM) *N. cucumeris* per 1.5 g and 887 ± 65.15 (SEM) *T. putrescentiae* mold mites per 1.5 g in the first trial and 243.00 ± 24.25 (SEM) *N. cucumeris* per 1.5 g and 575.00 ± 58.11 (SEM) *T. putrescentiae* mold mites per 1.5 g in the second. Similarly, the bran pile and sawdust pile treatments were also measured into 60 1.5 g portions and individually placed into the 59 mL soufflé cups. Bran material was also provided by BioBest Biological Systems and was comprised of the same material used to rear mites in the Amblyseius-Breeding-System, but did not contain mites. Sawdust (*i.e.*, small animal pet bedding) was purchased from a pet supply store in Okemos, MI, USA. We measured the breeder, bran, and sawdust piles into soufflé cups immediately prior to introduction in the experimental greenhouse. The 1.5 g pile size was chosen because the amount of mite-bran mixture in the fourth treatment, mini-sachets, was found to be approximately 1.5 g. The average number of mites released in breeder piles was determined by conducting Berlese funnel (Bioquip #2832, Rancho Dominguez, CA, USA) extractions on the 10 of the 60 remaining portions of each treatment that were measured into the soufflé cups at a Michigan State University Laboratory (East Lansing, MI, USA). No organisms were extracted from initial Berlese funnel samples of sawdust and bran piles in either trial.

Cups containing the breeder, bran, and sawdust material were poured from the soufflé cups into small piles on the soil surface of the barley beds in a grid pattern (as mentioned previously). Hanging sachets comprised of mini-sachets provided by BioBest Biological Systems were hung on card stakes at 16 cm above the soil in random locations on the same grid pattern that contained the other treatment piles. Similar to the other treatments, we also conducted Berlese funnel extractions of mini sachets and found that mini sachets contained 280.00 ± 25.23 (SEM) *N. cucumeris* per sachet and 2496.20 ± 65.45 (SEM) *T. putrescentiae* per sachet in the first trial and 482.00 ± 27.85 (SEM) *N. cucumeris* per sachet and 2980.20 ± 277.33 (SEM) *T. putrescentiae* per sachet in the second.

After all four treatments: breeder piles, bran piles, sawdust piles, and hanging sachets were in place on greenhouse bench blocks, Dalotia-System containers (containing 200 adult *D. coriaria*) were evenly distributed across the five blocks at a rate of approximately 40 beetles per block. We made a single release of *D. coriaria* beetles per trial. Dalotia-System material was provided by BioBest Biological Systems.

Amblyseius-Breeding-System and Dalotia-Systems were stored in a refrigerator at approximately 15 °C–16 °C for 24 h prior to introduction in the experiment. The duration of transit, arrival, and storage to introduction onto the benches was approximately 72 h.

Breeder piles (5), bran piles (5), sawdust piles (5) and hanging sachets (5) from each greenhouse bench block were randomly selected for collection at weekly intervals over a nine-week period (*n* = 20 per week). Each week, each of the treatment samples were individually placed into 59 mL plastic soufflé cups, and transported to a lab at Michigan State University for processing. Organisms in piles and hanging sachets were extracted into 95% ethanol using Berlese funnels. A 95% ethanol solution was used to preserve specimens for possible DNA analysis in the future [19]. Week 10 samples were not taken because sample collection ceased when mite densities extracted in previous weeks were low and when barley senesced. Organisms extracted from samples were counted using a dissecting microscope. We measured and compared densities of *D. coriaria*, *N. cucumeris*, and *T. putrescentiae* in the samples.

The number of *D. coriaria* in treatments were compared at weeks 1 through 7 to detect potential preferences of *D. coriaria* in these weeks. Data from weeks 8, 9, and 10 were not analyzed due to insufficient *D. coriaria* numbers. Data could not be normalized using transformations. Thus, Kruskal-Wallis rank sum tests were used to compare the overall numbers of *D. coriaria* in treatments and the numbers of *D. coriaria* in treatments at each week in R (R core development team 2011).

We analyzed the proportion change of *N. cucumeris* and *T. putrescentiae* population due to differing initial numbers of *N. cucumeris* and *T. putrescentiae* introduced in breeder piles and sachets (Figure 2). Proportion changes of populations were calculated by subtracting the initial mean mite species number from the weekly count of that species and dividing this difference by the initial mean mite species number. This calculation resulted in proportion increases and decreases of *N. cucumeris* and *T. putrescentiae* in samples that were translated into percentages. No *N. cucumeris* or *T. putrescentiae* were introduced in Bran and Sawdust piles thus this data was not included in the analysis. Data for weeks 8 and 9 in trial 1 were not analyzed due to insufficient numbers of *N. cucumeris* and *T. putrescentiae*. The proportions of *N. cucumeris* and *T. putrescentiae* recovered in treatments could not be normalized using transformations and were compared using Kruskal-Wallis rank sum tests in R (R core development team 2011).

### 2.2. Experiment Two—Materials and Methods

In this experiment, we monitored mite dispersal from breeder piles and sachets when *D. coriaria* was not present. The first trial of our second experiment was conducted in fall 2011 and the second trial of this experiment was repeated in winter 2011 in one 10 m × 7 m room of Michigan State University’s greenhouse in East Lansing, MI. The relative humidity and temperature in this room were regulated by ventstat (Micro Grow Greenhouse Systems, Inc., Temecula, CA, USA) and Sunne Controls thermostat (Detroit Radiant Products Co., Warren, MI, USA) set at 24 °C. The experiment was a completely randomized block design with seven blocks and one replicate per block.

Similar to the first experiment, we used barley as an experimental habitat to prevent unintentional rearing of greenhouse pests. Organic barley seed purchased from Johnny’s Selected Seeds was broadcast seeded at an approximate rate of 2 g per 930 cm^2^ onto the soil of 14 plastic containers 30 cm × 38 cm × 20 cm filled with Surefill brand Suremix Perlite potting soil comprised of peat moss and perlite. Barley was grown under natural light. Two containers of barley per each of the seven blocks were placed on a greenhouse bench; blocks were spaced 0.75 m between blocks and 0.3 m between containers in each block along the length of an 8 m greenhouse bench. Barley containers were irrigated twice per day until soil was saturated to a depth of approximately 2.5 cm using a hand-wand on the mist setting for the duration of the experiment.

A circular yellow sticky card (16.5 cm in diameter) was placed in the center of each of the barley beds. A circular piece of wax paper (10.5 cm in diameter) was placed in the center of each of the yellow sticky cards. Petri dishes (100 mm) filled with the same potting soil media used to grow barley were placed onto the wax paper, one dish per wax paper. Breeder piles were placed individually onto the soil of seven randomly selected Petri dishes. Sachets were placed individually onto the soil of the seven remaining Petri dishes.

Breeder pile and sachet treatments were prepared in a Michigan State University Laboratory prior to introduction in the experiment. Amblyseius-Breeding-System provided by BioBest Biological Systems was measured into 28 1.5 g piles and individually placed into 59 mL soufflé cups. Fourteen of the 28 piles were used in the experiment and initial Berlese funnel extractions were conducted on the remaining 14 1.5 g piles to determine the number of mites released. There was an average of 266 ± 15.25 (SEM) *N. cucumeris* per 1.5 g and 2803.46 ± 101.13 (SEM) *T. putrescentiae* mold mites per 1.5 g introduced in the first trial and 130.5 ± 7 (SEM) *N. cucumeris* per 1.5 g and 911.36 ± 30.24 (SEM) *T. putrescentiae* mold mites per 1.5 g introduced in the second trial. These piles were used to generate both treatments: breeder piles and sachets. We fabricated sachets by pouring one 1.5 g pile of the Amblyseius-Breeding-System that was previously measure into a 59 mL soufflé cup into an empty sachet. The opening on sachets into which the mite-bran mixture was poured (*i.e.*, not the same as the hole from which mites leave sachets) was sealed with a piece of clear plastic tape.

One week after breeder piles and sachets were introduced, yellow sticky cards containing mites that dispersed from the treatments were collected and sticky cards were replaced with new sticky cards containing wax paper as described previously. To collect the sticky cards, we lifted the Petri dishes containing either a breeder pile or sachet by hand, removed and replaced the used sticky card, and placed the Petri dish onto the center of the new sticky card. We collected and replaced sticky cards at weekly intervals for 9 weeks. The numbers of *N. cucumeris* and *T. putrescentiae* on yellow sticky cards were counted using a dissecting microscope. *Neoseiulus cucumeris* predatory mites data were normalized by transforming the number of *N. cucumeris* extracted from samples using log10 (x + 1) transformation. Normalized data were analyzed in R (R core development team 2011) using a two way ANOVA with factors: block, experimental treatment, and week. Main effects with *p* > 0.05 were removed from the model. Post hoc multiple comparisons were made using Tukey’s Honest Significant Difference.

*Tyrophagus putrescentiae* mold mites data could not be normalized using transformations. Therefore, significant differences between the numbers of *T. putrescentiae* in treatments were detected using weekly non-parametric Kruskal-Wallis rank sum tests in R (R core development team 2011).

## 3. Results

### 3.1. Experiment One Results

#### 3.1.1. *Dalotia coriaria* Extracted from Samples

**Figure 1:**
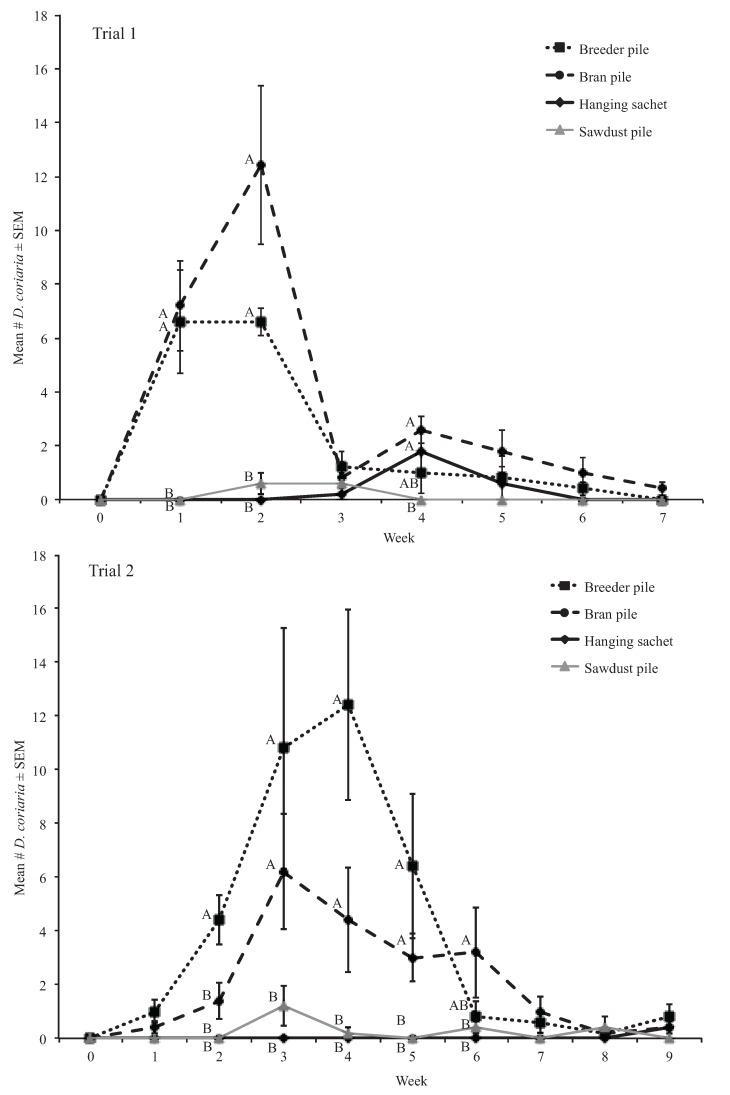
Mean number of *D. coriaria* extracted from treatments in trials 1 and 2. Different letters indicate significant differences among treatment means in the same sampling period (Kruskal-Wallis, α = 0.05).

Trial 1. We found significantly more *D. coriaria* in Bran (3.74 ± 0.85) and Breeder pile (2.37 ± 0.55) when compared with Sachet (0.37 ± 0.17) and Sawdust (0.17 ± 0.09) piles overall (*df* = 1, *H* ≥ 10.66, *p* < 0.01). Significant differences among treatments were found in weeks 1, 2, and 4. For all treatment comparisons using Kruskal-Wallis rank sum tests, there was one degree of freedom. In week 1, no *D. coriaria* were found in Sachets or Sawdust piles. Bran (7.20 ± 1.69) and Breeder piles (6.60 ± 1.91) had significantly higher *D. coriaria* numbers compared with Sachets or Sawdust piles (*H* = 7.81, *p* < 0.01) (Figure 1). Similarly, in week 2, no *D. coriaria* were found in Sachets. In the same week, very few *D. coriaria* were found in Sawdust piles (0.60 ± 0.40); Bran (12.40 ± 2.94) and Breeder piles (6.60 ± 0.51) contained significantly more *D. coriaria* than Sachets and Sawdust piles (*H* = 7.81, *p* < 0.01) (Figure 1). In week 4, no *D. coriaria* were found in Sawdust piles and although there were relatively few *D. coriaria* in Bran piles (2.60 ± 0.51) and Sachets (1.80 ± 0.86), Sawdust and Bran piles had significantly more *D. coriaria* than Sawdust piles (*H* = 7.81, *p* < 0.01; *H* = 5.58, *p* < 0.01, respectively) (Figure 1).

Trial 2. We found significantly more *D. coriaria* in Breeder (5.20 ± 0.83) and Bran (2.80 ± 0.35) piles than in Sachets (0 ± 0) and Sawdust (0.26 ± 0.07) piles overall (*df =*1, *H* ≥ 22.22, *p <* 0.01) (Figure 1). No *D. coriaria* were found in Sachets until week 9, when sachets contained an average of less than one *D. coriaria*. Significant differences among treatments were found in weeks 2, 3, 4, 5, and 6. For all treatment comparisons using Kruskal-Wallis rank sum tests, there was one degree of freedom. In week 2, no *D. coriaria* were found in Sachets or Sawdust piles, and Breeder piles contained significantly more *D. coriaria* (4.40 ± 0.93) than Bran piles (1.40 ± 0.68) (*H* = 4.06, *p* < 0.05), Sawdust piles and Sachets (*H* = 7.76, *p* < 0.01) (Figure 1). In week 3, Breeder (10.80 ± 4.49) and Bran (6.20 ± 2.15) piles contained significantly more *D. coriaria* than Sawdust piles (1.2 ± 0.73) (*H* = 4.87, *p* < 0.05; *H* = 4.06, *p* < 0.05, respectively), and Sachets (*H* = 7.76, *p* < 0.01) (Figure 1). In week 4, there was significantly more *D. coriaria* in Breeder (12.40 ± 3.54) and Bran (4.40 ± 1.94) piles than in Sawdust piles (0.20 ± 0.20) (*H* = 7.31, *p* < 0.01; *H* = 4.08, *p* < 0.05, respectively), and Sachets (*H* = 7.81, *p* < 0.01; *H* = 5.58, *p* < 0.05, respectively) (Figure 1). In week 5, significantly more *D. coriaria* was found in Breeder (6.40 ± 2.69) and Bran (3.00 ± 0.89) piles than Sawdust piles (*H* = 5.54, *p* < 0.05; *H* = 7.81, *p* < 0.01, respectively), and Sachets (*H* = 5.54, *p* < 0.05; *H* = 7.81, *p* < 0.01, respectively) (Figure 1).

#### 3.1.2. Weekly Proportion Change of *Neoseiulus cucumeris*

**Figure 2:**
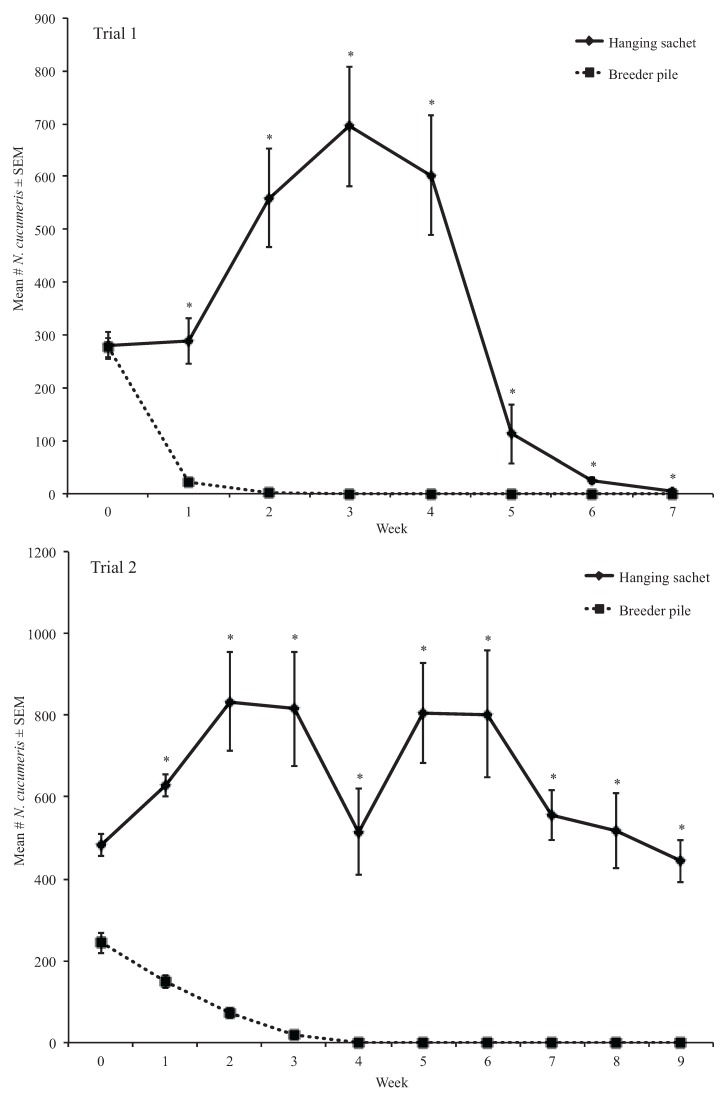
Mean number of *N. cucumeris* extracted from treatments in trials 1 and 2. * Means are significantly different among treatments in the same sampling period (Kruskal-Wallis, α = 0.05).

We found a significant effect of treatment on the proportion of *N. cucumeris* in breeder piles and hanging sachets in the first and second trials (*df* = 1, *H* = 43.02, *p* < 0.0001, *df* = 1, *H* = 67.02, *p* < 0.0001, respectively). The proportion of *N. cucumeris* in breeder piles was significantly less than those in hanging sachets in all weeks in both trials (*df* = 1, *H* ≥ 5.58, *p* < 0.05) (Table 1 and Table 2, Figure 2). Furthermore, there were proportionally fewer *N. cucumeris* in breeder piles than initially introduced in all weeks in both trials (Table 1 and Table 2, Figure 2). In contrast, the proportion of *N. cucumeris* in hanging sachets increased up to 148.00 ± 40.29 percent than the initial numbers of *N. cucumeris* introduced in the first trial and up to 66.98 ± 25.21 percent in the second trial (*df* = 1, *H* = 7.26, *p* < 0.01; *df* = 1, *H* = 7.26, *p* < 0.01, respectively) (Table 1 and Table 2, Figure 2).

**Table 1:** Percentage change ± SEM of mean *N. cucumeris* densities in treatments after introduction in experiment 1, trial 1. * indicates significant differences between treatments (Kruskal-Wallis, α = 0.05).

Week	Treatment	Percentage Change ± SEM	*H*-Value, *df* = 1	*p-*Value
1	Breeder pile	−92.49 ± 01.22	6.8182	0.0090 *
	Hanging sachet	02.86 ± 15.21		
2	Breeder pile	−99.71 ± 00.18	7.0313	0.0080 *
	Hanging sachet	99.71 ± 33.53		
3	Breeder pile	−99.86 ± 00.14	7.2581	0.0071 *
	Hanging sachet	148.00 ± 40.29		
4	Breeder pile	−99.86 ± 00.09	7.0313	0.0080 *
	Hanging sachet	115.07 ± 40.55		
5	Breeder pile	−99.93 ± 00.07	7.2581	0.0071 *
	Hanging sachet	−59.50 ± 20.04		
6	Breeder pile	−100.00 ± 00.00	7.7586	0.0053 *
	Hanging sachet	−91.14 ± 01.65		
7	Breeder pile	−100.00 ± 00.00	5.5814	0.0182 *
	Hanging sachet	−98.36 ± 00.88		

**Table 2:** Percentage change ± SEM of mean *N. cucumeris* densities in treatments after introduction in experiment 1, trial 2. * indicates significant differences between treatments (Kruskal-Wallis, α = 0.05).

Week	Treatment	Percentage Change ± SEM	*H*-Value*, df* =1	*p-*Value
1	Breeder pile	−38.90 ± 06.78	6.8182	0.0090 *
	Hanging sachet	30.53 ± 05.72		
2	Breeder pile	−70.23 ± 04.92	6.8182	0.0090 *
	Hanging sachet	72.87 ± 25.19		
3	Breeder pile	−92.52 ± 03.72	6.8598	0.0088 *
	Hanging sachet	69.02 ± 28.86		
4	Breeder pile	−99.84 ± 00.10	7.0313	0.0080 *
	Hanging sachet	06.89 ± 22.15		
5	Breeder pile	−99.92 ± 00.08	7.2581	0.0071 *
	Hanging sachet	66.98 ± 25.21		
6	Breeder pile	−100.00 ± 00.00	7.7586	0.0053 *
	Hanging sachet	66.49 ± 32.02		
7	Breeder pile	−99.92 ± 00.08	7.2581	0.0071 *
	Hanging sachet	15.47 ± 12.87		
8	Breeder pile	−99.92 ± 00.08	7.2581	0.0071 *
	Hanging sachet	07.55 ± 19.15		
9	Breeder pile	−100.00 ± 00.00	7.7586	0.0053 *
	Hanging sachet	−08.13 ± 10.54		

#### 3.1.3. Weekly Proportion Change of *Tyrophagus putrescentiae*

**Figure 3:**
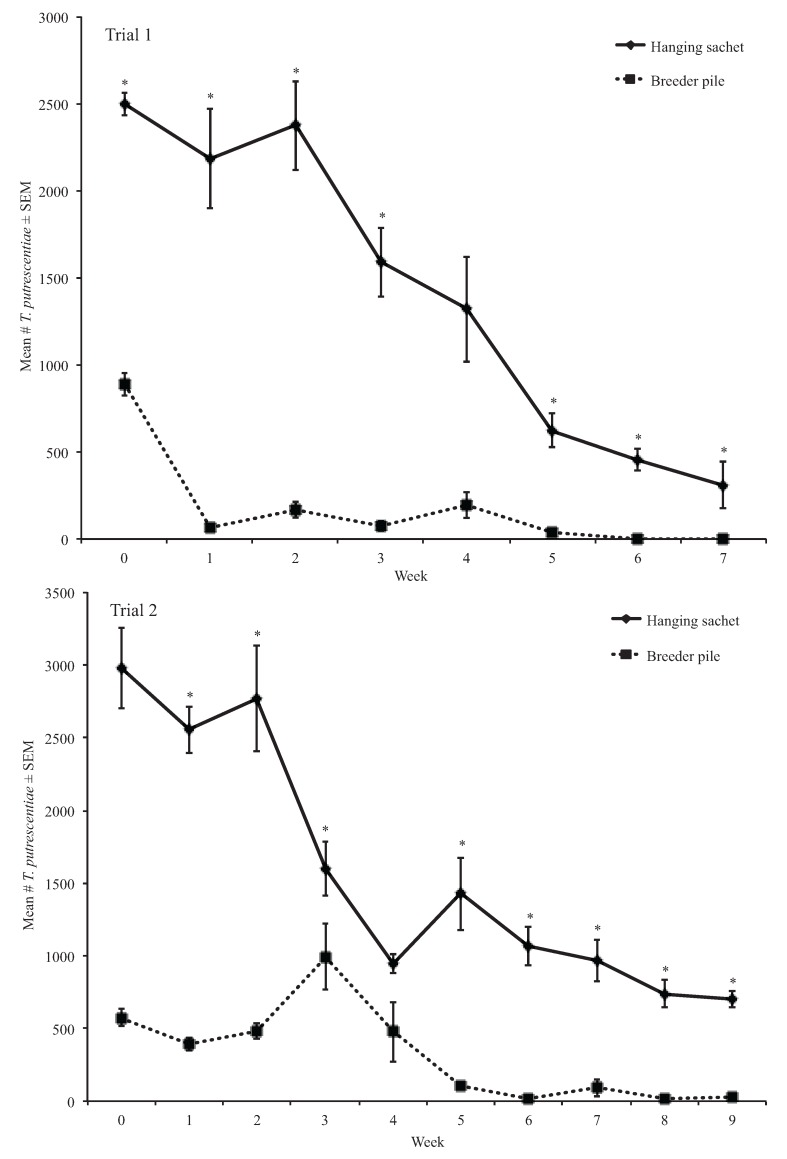
Mean number of *T. putrescentiae* extracted from treatments in trials 1 and 2. * Means are significantly different among treatments in the same sampling period (Kruskal-Wallis, α = 0.05).

We found a significant effect of treatment on the proportion of *T. putrescentiae* in breeder piles and hanging sachets (*df* = 1, *H* = 32.68, *p* < 0.05). The proportion of *T. putrescentiae* in breeder piles was significantly less than the proportion of *T. putrescentiae* in hanging sachets in weeks 1, 2, 3, 5, and 6 in the first trial and in weeks 5, 6, 8, and 9 in the second trial (*df* = 1, *H* ≥ 6.82, *p* < 0.05) (Table 3 and Table 4, Figure 3). In week 3, the proportion of *T. putrescentiae* in breeder piles was significantly greater than in hanging sachets (*df* = 1, *H* = 6.82, *p* < 0.01) (Table 3 and Table 4, Figure 3).

**Table 3:** Percentage change ± SEM of mean *T. putrescentiae* densities in treatments after introduction in experiment 1, trial 1. * indicates significant differences between treatments (Kruskal-Wallis, α = 0.05).

Week	Treatment	Percentage Change ± SEM	*H*-Value*, df* =1	*p-*Value
1	Breeder pile	−92.97 ± 00.58	6.8182	0.0090 *
	Hanging sachet	−12.41 ± 11.48		
2	Breeder pile	−81.10 ± 05.12	6.8182	0.0090 *
	Hanging sachet	−04.89 ± 10.26		
3	Breeder pile	−91.61 ± 02.93	6.8182	0.0090 *
	Hanging sachet	−36.24 ± 07.97		
4	Breeder pile	−78.02 ± 08.56	3.1527	0.0758
	Hanging sachet	−47.08 ± 12.13		
5	Breeder pile	−95.49 ± 01.81	6.8182	0.0090 *
	Hanging sachet	−75.04 ± 03.97		
6	Breeder pile	−97.41 ± 01.90	6.8182	0.0090 *
	Hanging sachet	−81.68 ± 02.58		
7	Breeder pile	−100.00 ± 00.00	7.7586	0.0053 *
	Hanging sachet	−87.63 ± 05.44		

**Table 4:** Percentage change ± SEM of mean *T. putrescentiae* densities in treatments after introduction in experiment 1, trial 1. * indicates significant differences between treatments (Kruskal-Wallis, α = 0.05).

Week	Treatment	Percentage Change ± SEM	*H*-Value*, df* =1	*p-*Value
1	Breeder pile	−32.38 ± 07.85	3.1527	0.0758
	Hanging sachet	−14.23 ± 05.39		
2	Breeder pile	−16.24 ± 09.11	0.2727	0.6015
	Hanging sachet	−06.99 ± 12.23		
3	Breeder pile	72.31 ± 39.61	6.8182	0.0090 *
	Hanging sachet	−46.25 ± 6.30		
4	Breeder pile	−17.15 ± 35.82	0.8836	0.3472
	Hanging sachet	−68.29 ± 02.11		
5	Breeder pile	−81.74 ± 04.17	6.8182	0.0090 *
	Hanging sachet	−52.14 ± 08.39		
6	Breeder pile	−96.31 ± 01.59	6.8598	0.0088 *
	Hanging sachet	−64.30 ± 04.44		
7	Breeder pile	−83.83 ± 10.36	2.4545	0.1172
	Hanging sachet	−67.65 ± 04.84		
8	Breeder pile	−97.88 ± 01.50	6.8182	0.0090 *
	Hanging sachet	−75.28 ± 03.18		
9	Breeder pile	−95.44 ± 03.15	6.8182	0.0090 *
	Hanging sachet	−76.48 ± 01.91		

### 3.2. Experiment Two Results

#### 3.2.1. *Neoseiulus cucumeris* Predatory Mites

**Figure 4:**
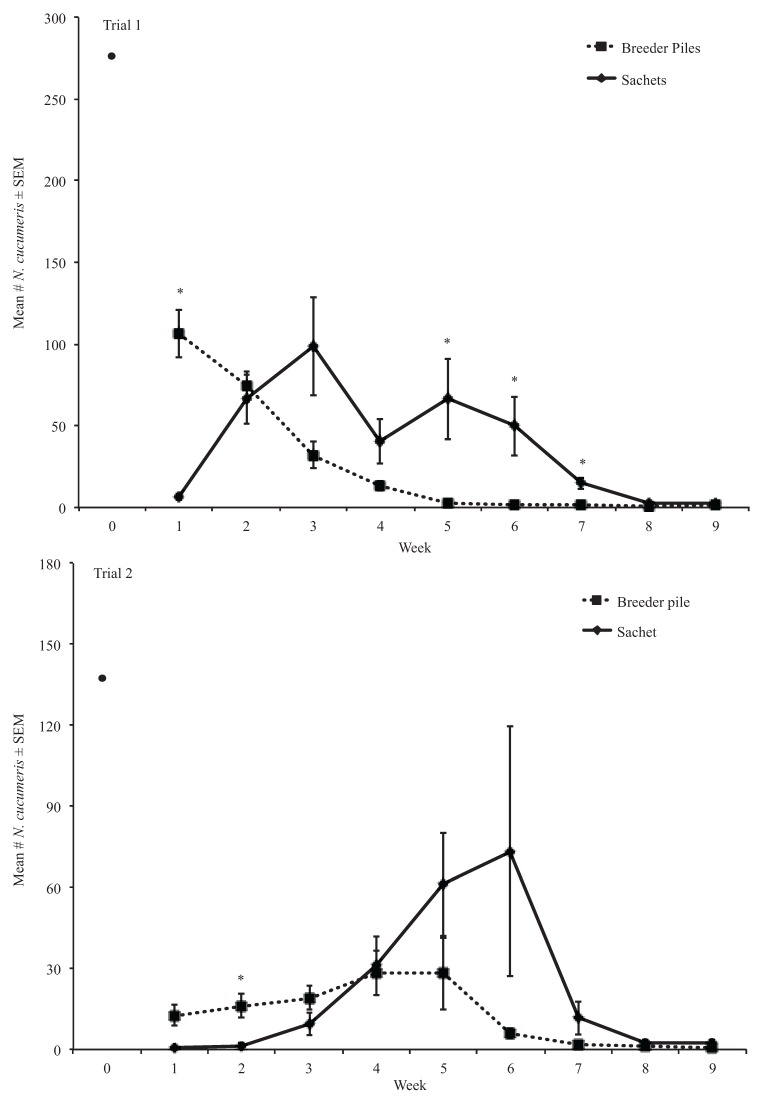
Mean number of *N. cucumeris* that dispersed from treatments in trials 1 and 2. * Means are significantly different among treatments in the same sampling period (Tukey’s HSD, α = 0.05).

In the first trial of this second experiment, we found that significantly more *N. cucumeris* dispersed from sachets (38.59 ± 6.45) than from breeder piles (25.94 ± 5.05) overall (Figure 4). The ANOVA test indicated significant treatment, week, and the treatment by week interaction effects (*df* = 1, *F-value* = 22.35, *p* < 0.01; *df* = 8, *F-value* = 29.28, *p* < 0.01; *df* = 8, *F-value* = 13.68, *p* < 0.01, respectively) (Figure 4). Significant week effects were observed among the early weeks (e.g., 1–4) and later weeks (e.g., 5–9) (Figure 4). The treatment by week interaction was significant in weeks 1, 5, 6, and 7 (Figure 4). In week 1, significantly more mites dispersed from breeder piles than from sachets (Figure 4). Significantly more *N. cucumeris* was observed dispersing from sachets than from breeder piles in weeks 5, 6, and 7 (Figure 4).

In the second trial of this experiment, the ANOVA test indicated significant effects of block, week, and the treatment by week interaction (*df* = 6, *F-value* = 2.38, *p* < 0.05; *df* = 8, *F-value* = 14.76, *p* < 0.01; *df* = 8, *F-value* = 6.58, *p* < 0.01, respectively) (Figure 4). Significant week effects were observed among early weeks (e.g., 1–4) and later weeks (e.g., 5–9) (Figure 4). A significant interaction effect was observed in week 2 where more *N. cucumeris* dispersed from breeder piles (13.20 ± 5.64) than from sachets (1.60 ± 1.60) (Figure 4).

**Figure 5:**
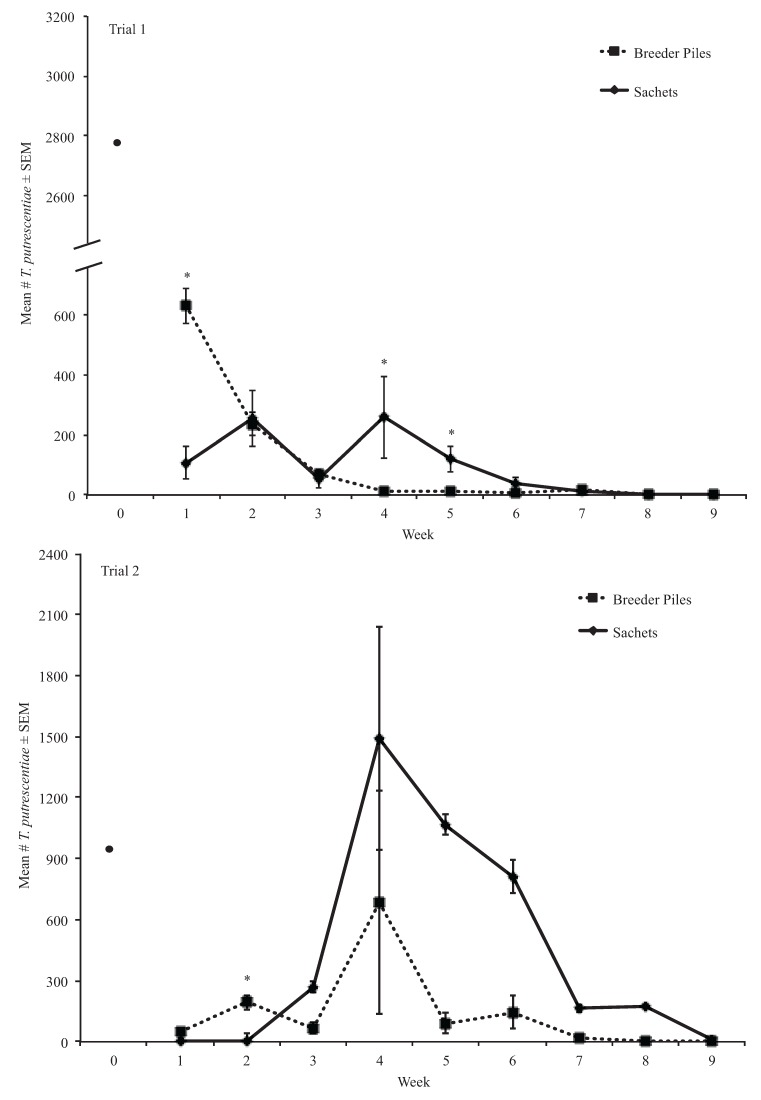
Mean number of *T. putrescentiae* that dispersed from treatments in trials 1 and 2. * Means are significantly different among treatments in the same sampling period (Kruskal-Wallis, α = 0.05).

#### 3.2.2. *Tyrophagus putrescentiae* Mold Mites

We did not find a significant treatment effect on the number of *T. putrescentiae* that dispersed from breeder piles or sachets in the first or second trials using a Kruskal-Wallis rank sum test. In the first week of the first trial, significantly more *T. putrescentiae* dispersed from breeder piles (109.29 ± 26.20) than from sachets (94.25 ± 22.63) (*df* = 1, *H* = 9.80, *p* < 0.01) (Figure 5). In weeks 4 and 5, significantly more *T. putrescentiae* mites dispersed from sachets than from breeder piles (*df* = 1, *H* = 4.45, *p* < 0.05; *df* = 1, *H* = 5.60, *p* = 0.02, respectively) (Figure 5).

In the first and second weeks of the second trial, significantly more *T. putrescentiae* dispersed from breeder piles (40 ± 22.36 and 203.00 ± 46.81, respectively) than from sachets (4.20 ± 0.97 and 2.60 ± 1.44, respectively) (*df* = 1, *H* = 5.03, *p* < 0.05; *df* = 1, *H* = 9.82, *p* < 0.01, respectively) (Figure 5). In week 5, significantly more *T. putrescentiae* dispersed from sachets (13.50 ± 857.77) than from breeder piles (127.80 ± 66.17) (*df* = 1, *H* = 5.60, *p* < 0.05) (Figure 5).

## 4. Discussion

Methods for open rearing *N. cucumeris* in greenhouses include the use of breeder piles and sachets. These open rearing systems are comprised of a mixture of bran, *T. putrescentiae* mold mites, and *N. cucumeris* predatory mites that are either applied in piles to the soil (*i.e.*, breeder piles) or placed in a paper envelope (*i.e.*, sachets) that can be hung in the plant canopy. Breeder piles place *N. cucumeris* onto the soil where they are vulnerable to predation by *D. coriaria* [16]. Increasing habitat complexity has demonstrated positive results for reducing intraguild predation [6]. We used sachets to increase habitat complexity and reduce intraguild predation between *N. cucumeris* and *D. coriaria*. Our results showed that sachets were an effective tool for delaying, reducing, and in some cases eliminating the invasion of *N. cucumeris* mite-bran material by *D. coriaria*. Therefore, using sachets rather than breeder piles in greenhouses where *D. coriaria* have been released would be a better open rearing approach.

Understanding reasons why *D. coriaria* invade breeder piles is the first step for mitigating invasion. Several possible reasons include: foraging for *N. cucumeris and T. putrescentiae* as prey resources, foraging for other prey species utilizing the pile material, foraging for the bran and fungus itself, or seeking a physical shelter or oviposition site. We hypothesized that *D. coriaria* were attracted to breeder piles for the prey resources they harbor—*i.e.*, predator and fungus mites or other arthropods or fungi growing on the bran.

Bran piles did not initially contain mites or other organisms but were invaded by other mites, collembola, and dipterans as well as *D. coriaria* within one week after introduction to the soil. Bran and the fungi that grow on it are likely attractive food sources for these organisms. *Dalotia coriaria* are polyphagous [15,20] and it is likely that *D. coriaria* were consuming bran, fungi, and arthropods in the piles.

Few *D. coriaria* were recovered from sawdust piles indicating that sawdust is less attractive to *D. coriaria* than bran and breeder piles. Therefore, structural aspects of the piles were not key factors influencing *D. coriaria* presence in bran and breeder piles. In general, fewer organisms were observed in sawdust than in other treatments. It is likely that fewer organisms were found in sawdust because celluloses of woody plant materials are difficult for many arthropods to digest [21] making the piles an inadequate food source or location for food.

The presence of more *D. coriaria* in bran and breeder piles than in sawdust piles provided evidence that *D. coriaria* were probably invading piles for food resources rather than for the physical structure of piles. Therefore, mitigating invasion of *D. coriaria* into the bran and breeder pile material is unlikely a simple scenario. Providing a barrier for breeder pile material that would allow mite dispersal, but prevent *D. coriaria* from entering is one possibility. This could be achieved by placing sachets on plug trays where young plants cannot support a hanging sachet. On the other hand, developing an open rearing system for *D. coriaria* by placing bran piles on the soil that attracts organisms for *D. coriaria* consumption could be promising and would be an interesting focus for future research.

Sachets and breeder piles are used to prolong releases of predatory mites for management of mite pests and thrips in greenhouse crops. With the exception of Shipp and Wang [11], there has been little published work on the number of mites that are produced by and dispersing from these open rearing systems over time. Because initial densities of *N. cucumeris* and *T. putrescentiae* introduced in sachets and breeder piles were dissimilar, we compared proportions of these mites. Our results show that sachets could have proportionally more *N. cucumeris* than breeder piles for up to nine weeks and possibly longer as there were still *N. cucumeris* present in sachets when the second trial of the first experiment ended. The differences in the numbers of *N. cucumeris* observed in sachets in the first and second trials could be due to the presence of *D. coriaria* in the sachets in weeks three through five in the first trial (Figure 2). Furthermore, the initial numbers and ratios of predator and prey mites in the sachets differed between trials and could have played a role in the numbers of *N. cucumeris* that were produced by the sachets overtime.

Proportions of *T. putrescentiae* in sachets and breeder piles demonstrated mostly decreasing trends over time (Table 3 and Table 4, Figure 3). Throughout the experiment, sachets supported proportionally more mites compared with breeder piles. Maintaining *T. putrescentiae* populations is essential for providing *N. cucumeris* with a food source for renewing populations of *N. cucumeris* in these open rearing systems.

Our second study was conducted in a Michigan State University greenhouse where *D. coriaria* had not been released to compare the numbers of mites dispersing from sachets and breeder piles in the absence of *D. coriaria*. The presence of *D. coriaria* could influence mite dispersal in at least three ways including negatively impacting the number of mites in breeder pile or sachet open rearing systems by feeding on the mites, disturbing the open rearing systems resulting in mite migration, and/or causing mites to leave to avoid predation. In both trials, sachets provided prolonged dispersal of both *N. cucumeris* predators and *T. putrescentiae* mold mites compared with breeder piles. Additionally, more *N. cucumeris* and *T. putrescentiae* dispersed from breeder piles than sachets within the first and/or second week after introduction, and the number of *N. cucumeris* that dispersed from sachets over time was greater than the number that dispersed from breeder piles. This result suggests that growers that wish to utilize both natural enemies can minimize intraguild predation by using sachet style release methods.

Although the general trends of mite dispersal were similar in both trials of the second experiment, there was variability in the pattern of dispersal between trials. Some factors which may have led to this variability include differences in: photoperiod, temperature, initial numbers of mites introduced, ratios of initial predator and prey mites introduced, and disturbance to treatments by cockroaches. The first trial was conducted from mid-August to early October when photoperiod was longer and temperatures were higher, whereas the photoperiod was shorter and temperatures were lower from early December to early February in the second. Mite development and reproduction was likely advanced or delayed due to higher or lower temperatures in these trials. Faster reproduction and population growth could have resulted in a higher proportion of mites dispersing initially in the first trial or in contrast, a delayed mite dispersal when population growth is slower. Initial numbers of mites in the first trial were two-fold more *N. cucumeris* predatory mites and three-fold more *T. putrescentiae* mold mites than in the second trial. Differences in the numbers of mites introduced may have also contributed to slower population growth. Furthermore, cockroaches disturbed experimental units, chewed through the paper sachets, and scattered breeder piles material in the second trial. Cockroaches have not previously been observed disturbing mite-bran material, but have been reported as greenhouse pests [22,23].

The findings of our experiments have generated many opportunities for future research. We observed the numbers of mites produced in open rearing systems when *D. coriaria* was present and mite dispersal from open rearing units in the absence of *D. coriaria*. Research that observes both the numbers of mites produced by and that disperse from breeder piles and sachets would provide better insight to optimize the numbers of mites introduced for desired mite production and dispersal rates. The influence of climatic conditions such as photoperiod, temperature, and humidity on the numbers of mites produced and that disperse should be considered in such research. Optimal initial densities of predatory mites and mold mites to prolong mite production from open rearing systems should also be investigated. Our research has also opened opportunities for investigating open rearing systems for *D. coriaria*. Introducing bran piles that provide fungi and lure arthropods for *D. coriaria* consumption may be a promising tool. Furthermore, using bran piles as a means of monitoring soil arthropod presence is another possibility for research. Addressing these topics in future experiments would improve the use of open rearing systems in greenhouses.

## 5. Conclusions

The results from our first experiment showed that sachets protected *N. cucumeris* from intraguild predation and competition by *D. coriaria*. Therefore, *N. cucumeris* sachets, not breeder piles, should be used in greenhouses that also release *D. coriaria* in biological pest management programs. Furthermore, sachets produced and maintained more *N. cucumeris* and *T. putrescentiae* than breeder piles. The duration of mite production in sachets was also greater compared to breeder piles. These results indicate that fewer releases of *N. cucumeris* may be needed if sachets are used. Results from our second experiment showed that mite dispersal from breeder piles and sachets differed. More mites dispersed from breeder piles than sachets in earlier weeks and the opposite was true in later weeks. Therefore, breeder piles may be more appropriate for “quick-releases” of *N. cucumeris* whereas slow-release sachets are true to their name. Although both breeder pile and sachet open rearing systems should be introduced preventatively (*i.e.*, when pest densities are low), our results indicate that introductions of sachets could be made sooner than breeder piles to compensate for delayed mite dispersal.
